# Quality and resilience of clinical laboratories in Rwanda: a need for sustainable strategies

**DOI:** 10.1080/16549716.2024.2358633

**Published:** 2024-06-03

**Authors:** Vincent Rusanganwa, Innocent Nzabahimana, Magnus Evander

**Affiliations:** a Department of Clinical Microbiology, Umeå University, Umeå, Sweden; b Department of Health Workforce, Ministry of Health, Kigali, Rwanda

**Keywords:** Quality improvement, SLMTA, laboratory assessment, health system, intervention

## Abstract

**Background:**

Quality healthcare is a global priority, reliant on robust health systems for evidence-based medicine. Clinical laboratories are the backbone of quality healthcare facilitating diagnostics, treatment, patient monitoring, and disease surveillance. Their effectiveness depends on sustainable delivery of accurate test results. Although the Strengthening Laboratory Management Towards Accreditation (SLMTA) programme has enhanced laboratory quality in low-income countries, the long-term sustainability of this improvement remains uncertain.

**Objective:**

To explore the sustainability of quality performance in clinical laboratories in Rwanda following the conclusion of SLMTA.

**Methods:**

A quasi-experimental design was adopted, involving 47 laboratories divided into three groups with distinct interventions. While one group received continuous mentorship and annual assessments (group two), interventions for the other groups (groups one and three) ceased following the conclusion of SLMTA. SLMTA experts collected data for 10 years through assessments using WHO’s StepwiseLaboratory Quality Improvement Process Towards Accreditation (SLIPTA) checklist. Descriptive and t-test analyses were conducted for statistical evaluation.

**Results:**

Improvements in quality were noted between baseline and exit assessments across all laboratory groups (mean baseline: 35.3%, exit: 65.8%, *p* < 0.001). However, groups one and three experienced performance declines following SLMTA phase-out (mean group one: 64.6% in reference to 85.8%, *p* = 0.01; mean group three: 57.3% in reference to 64.7%, *p* < 0.001). In contrast, group two continued to enhance performance even years later (mean: 86.6%compared to 70.6%, *p* = 0.03).

**Conclusion:**

A coordinated implementation of quality improvement plan that enables regular laboratory assessments to pinpoint and address the quality gaps is essential for sustaining quality services in clinical laboratories.

## Paper context

- **Main findings:** We found that continuous laboratory quality improvement was achieved by laboratories that kept up with regular follow-ups, as opposed to those which phased out these followups prematurely.
- **Added knowledge:** This study has affirmed the necessity of maintaining mentorship and conducting regular quality assessments until requisite quality routines are established to sustain laboratory quality services.
- **Global health impact for policy and action:** These findings emphasise the significance of instituting a laboratory quality plan, with regular assessments, as policy directives to uphold and enhance quality standards, which benefits both local and global communities, given the pivotal role of laboratories in patient treatment, disease prevention, and surveillance

## Background

In an era of advancing healthcare systems and global health challenges, sustainable quality in clinical laboratories plays a pivotal role in ensuring the delivery of quality healthcare services [1,2]. Clinical laboratories are essential components of healthcare systems worldwide, facilitating disease diagnosis, monitoring, and research [3–6]. However, their effectiveness and impact on public health are critically dependent on the quality of services they provide [4,7]. Quality assurance in clinical laboratories is not only a local concern but also a global imperative, with far-reaching consequences for patient outcomes, disease surveillance, and research endeavours [8,9]. As healthcare systems grapple with challenges, such as increasing patient demands, emerging and re-emerging diseases, emerging technologies, and evolving regulatory standards, the sustainability of clinical laboratory quality emerges as a critical concern [10,11].

Sustainable clinical laboratory quality is a topic of growing importance in our ever-evolving healthcare landscape [2]. As the demand for diagnostic services continues to surge, laboratories are confronted with the imperative to balance increased throughput with uncompromising accuracy and reliability [12,13]. This challenging equilibrium necessitates a holistic and systematic approach that encompasses every facet of laboratory operations, including regulatory standards compliance [14].

The ongoing global challenges related to healthcare, such as the COVID-19 pandemic and other emerging and re-emerging infectious diseases, have underscored the need for resilient clinical laboratory systems that can adapt and thrive in times of crisis [15,16]. Beyond these acute crises, long-term sustainability is equally critical to ensure that laboratories can continue to meet the evolving demands of a rapidly advancing field of medicine.

Low and middle-income countries (LMICs) often characterised by resource constraints, limited infrastructure, and systemic challenges, face obstacles in maintaining the quality of their clinical laboratory services [17–19]. The availability and sustainability of high-quality clinical laboratory services remain a persistent challenge [20]. The intersection of these limited resources creates an obstacle to achieving sustainable and consistent laboratory quality in these countries [19,20].

The Strengthening Laboratory Management Toward Accreditation (SLMTA) programme plays a crucial role in enhancing the quality of clinical laboratories in LMICs [21]. By providing structured training, mentorship, and assessment tools, SLMTA empowers laboratories to meet international quality standards, thereby improving the accuracy and reliability of diagnostic services crucial for patient care and disease surveillance [21]. This capacity-building initiative has been instrumental in strengthening healthcare systems in resource-constrained settings, ultimately contributing to better health outcomes [22]. Similar to many other limited-resource countries, the SLMTA programme has demonstrated a positive impact on clinical laboratories in Rwanda by enhancing their quality performance [23,24].

The healthcare landscape in Rwanda has undergone remarkable transformation over the last two decades, making substantial strides in improving the quality of healthcare services and overall health outcomes for its population [25]. A cornerstone of this transformation has been the development and expansion of clinical laboratory services alongside the health facilities to improve healthcare geographical accessibility [23]. However, as Rwanda’s healthcare system continues to evolve, the sustainability of clinical laboratory quality is becoming a critical concern, demanding rigorous assessment and strategic planning [24]. This paper focuses on Rwanda as a case study to explore the sustainability of quality in clinical laboratories within the context of both global and Rwandan healthcare priorities.

## Methods

### Study settings

This study included 47 clinical laboratories in Rwanda, five of which serve as referral laboratories and assume a supervisory role regarding the other laboratories in their catchment areas. These five referral laboratories include the National Reference Laboratory (NRL), which oversees laboratory operations throughout the country. The remaining 42 laboratories operate at the district hospitals or at newly upgraded provincial, referral, and level 2 teaching hospitals which are still functioning as district hospitals as of 2024. All these 42 are referred to as ‘district laboratories’ in this paper.

### Study design

This study used a quasi-experimental design. In total, 49 clinical laboratories in Rwanda were enrolled in SLMTA since its inception in 2010, and 47 of them were considered in this study as the two remaining laboratories did not undergo either the exit or the follow-up assessment. To evaluate the sustainability of the quality performance of these laboratories, laboratories were categorized into three groups with different interventions. The first group was comprised of five referral laboratories, referred to as R1 to R5, enrolled in 2010; the second group consisted of five district laboratories, referred to as D1 to D5, enrolled in 2011; and the third group was comprised the 37 remaining district laboratories, referred to as 1 to 37, enrolled in different cohorts from 2013 to 2017. The enrollment in different cohorts was intended to result in smaller and more manageable groups for trainings and mentorship.

SLMTA interventions common to all three groups consisted of baseline, exit, follow-up assessments, and training of laboratory personnel in the Laboratory Quality Management System (LQMS) as well as mentorship in quality improvement projects implementation between baseline and exit assessments. These SLMTA interventions were conducted during the first year between baseline and exit assessments. Follow-up assessments were conducted for laboratory groups one and two after one year of exit assessments. However, for group three laboratories, as they were enrolled in SLMTA in different years and in different cohorts, this assessment was conducted in 2019, with varying intervals from the exit assessments.

The second group, in addition to the common interventions, continued to be mentored from 2011 to 2016, and had performance-based financial incentives from 2011 to 2015 [26], and annual assessments until 2019, with the exception of two laboratories which benefited from assessments in 2020. Mentorship consisted of regular supportive supervision and coaching by experts in laboratory quality improvement to identify and address any gap in quality. Financial incentives were decreased or not provided depending on the level of decrease in performance [26]. Laboratories in groups one and three did not receive any additional financial schemes beyond their ordinary budgets, except for the NRL, which received the financial support that was not based on its performance and which was not evaluated annually. Moreover, the first group had an additional assessment in 2017 pertaining to research [23] and all laboratory groups were assessed in 2019, except one laboratory in first group. The Table 1 illustrates different interventions per laboratory group and year of intervention.

**Table 1.** Interventions per laboratory group between 2010 and 2019 (X indicates the year of intervention).

Laboratory groups & Interventions	2010	2011	2012	2013	2014	2015	2016	2017	2018	2019
**Group one laboratories (R1-R5) *n*=5**										
Baseline	X									
Training in LQMS	X									
Mentorship	X	X	X							
Exit		X								
Follow-up			X							
Other assessments								X		X
**Group two laboratories (D1-D5) *n*=5**										
Baseline		X								
Training in LQMS		X								
Financial incentives		X	X	X	X	X				
Mentorship		X	X	X	X	X	X			
Exit			X							
Follow-up				X	X	X	X	X	X	X
**Group three laboratories (1–37) *n*=37**										
**Cohort one (# 5)**										
Baseline				X						
Training in LQMS				X						
Mentorship				X						
Exit					X					
Follow-up										X
**Cohort two (# 8)**										
Baseline					X					
Training in LQMS					X					
Mentorship					X					
Exit						X				
Follow-up										X
**Cohort three (# 9)**										
Baseline						X				
Training in LQMS						X				
Mentorship						X				
Exit							X			
Follow-up										X
**Cohort four (# 9)**										
Baseline							X			
Training in LQMS							X			
Mentorship							X			
Exit								X		
Follow-up										X
**Cohort five (# 6)**										
Baseline								X		
Training in LQMS								X		
Mentorship								X		
Exit									X	
Follow-up										X

### Data collection

In all laboratories, the Stepwise Laboratory Quality Improvement Process Towards Accreditation (SLIPTA) checklist, a tool provided by the World Health Organization (WHO), Africa Region [27] was used for every assessment. Data were collected across the years indicated in Table 1 by experts in SLMTA and were obtained from NRL, except those from 2017 for referral laboratories that were collected for our previous study [23].

Baseline, exit, and follow-up data, for the group one laboratories, were collected in 2010, 2011, and 2012, respectively and other data were collected via 2017 and 2019 assessments (Table 1). The R1 and R5 were internationally accredited in 2020 and 2021, respectively. Their years of ISO 15,189 (International Organisation for Standardisation) accreditation were also taken into consideration. A score of 100% was purposely assigned to each of these laboratories for their specific year of accreditation. Since R1 was not assessed in 2019 and was accredited in 2020, we retained the score from 2017 for this laboratory in 2019, and similarly, for R5 in 2020, we retained the score from 2019. These scores were arranged in such a way as to create a continuous graph. However, those scores not generated through SLIPTA checklist were not considered in the statistical analysis.

Data from the five laboratories in the second group (D1-D5) were collected every year from 2011 to 2019 through continuous assessments (Table 1) and two of them had had additional assessments in 2020 before the COVID-19 restrictions began in Rwanda. The data for the third group (1 to 37) were collected along with their baseline and exit assessments conducted in various years from 2013 to 2017, depending on the cohort enrolled in the SLMTA programme. This laboratory group underwent a follow-up assessment in 2019 (Table 1).

### Data analysis

To illustrate the evolution of quality performance over the years, we relied on percentage scores. It is important to note that the WHO grading scale uses a score ranging from zero to five stars, corresponding to specific performance score percentage intervals: <55, (≥55 to < 65), (≥65 to < 75), (≥75 to < 85), (≥85 to < 95), and ≥ 95, respectively. Utilizing percentages allowed us to capture nuanced variations in performance for each assessment, while star scores are based on these percentage intervals. Total performance was calculated as the sum of scores for the twelve Quality System Essentials (QSEs) assessed in each laboratory. Trends in performance over the years were graphically depicted for comparison.

To statistically compare the performance mean differences between different assessments, the test of student (T-test) was applied and a *p* value <0.05 was considered statistically significant. Quality performance between baseline and exit assessments was tested for 47 laboratories altogether, while this performance for other different assessments was tested within each specific laboratory group based on their different interventions. The performance at exit assessment was considered as the reference to measure continuous improvement in following assessments, except for the group one laboratories, where the follow-up assessment was used as reference as they continued to have mentorship until the follow-up assessment rather than the exit assessment. Comparison of subsequent performances to the exit assessments was based on confirmed quality improvements observed at the exit assessments, along with the cessation of intervention for most of the laboratories. Furthermore, using the exit point as a benchmark enabled the underscore of any differences between the laboratory group undergoing continuous interventions and the other two laboratory groups lacking such interventions.

### Ethical considerations

The research proposal received approval from the Rwanda National Ethics Committee, with references 0059/RNEC/2017, 111/RNEC/2018, and 096/RNEC/2020, as well as endorsement from the Rwandan Ministry of Health. It is important to note that this study did not involve human participants but instead focused on the laboratory systems.

## Results

In this study, we present sustainability patterns regarding the quality performance of clinical laboratories in Rwanda following the implementation of the SLMTA programme. The SLMTA programme was effective, as demonstrated by the results of the baseline and exit assessments. At the baseline, 98% (46 out of 47) of laboratories received a zero-star rating. However, within one year of implementation, significant progress was observed, with 89% (42 out of 47) of laboratories achieving at least a one-star rating. Although the five remaining laboratories made considerable percentage improvements, they fell short of reaching the one-star threshold (Table 2). The quality improvement was confirmed by a difference in mean scores between the two assessments, which is highly statistically significant at a 95% confidence interval (*p* < 0.001) (Table 3). Nevertheless, it is noteworthy that most laboratories experienced a decline in their quality performance following the cessation of SLMTA intervention, signifying sustainability challenges. In contrast, the five laboratories in group two which continued to receive mentorship and underwent continuous annual assessments exhibited a consistent and progressive increase in their quality performance, with a baseline mean (SD) of 29.4% (13.0), an exit mean (SD) of 70.6% (7.3), *p* < 0.001, and nine years later, a mean compared to exit of 86.6% (5.2), *p* = 0.03 with a 95% confidence interval.

**Table 2.** Laboratory groups quality performance in star rating from baseline, exit, and follow-up assessments (n = 47).

Laboratory groups assessments	0 star	1 star	2 stars	3 stars	4 stars	5 stars
**Group one laboratories** **(R1-R5) *n*=5**						
Baseline	4	1				
Exit		1	2	2		
Follow-up				2	3	
**Group two laboratories** **(D1-D5) *n*=5**						
Baseline	5					
Exit		1	2	2		
Follow-up				2	3	
**Group three laboratories** **(1–37) *n*=37**						
Baseline	37					
Exit	5	10	17	4	1	
Follow-up	9	22	4	2

**Table 3.** Quality performance changes in three laboratory groups with different interventions.

Laboratory groups		Year of assessment	Mean (SD)	[95% CI]	*p* value
**All laboratory groups(*n*=47)**					
	Baseline	Different years	35.3(10.1)	[32.3–38.3]	
	Exit	Different years	65.8(9.2)	[63.2–68.5]	<0,001
**Laboratory group one (*n*=5)**					
	Baseline	2010	43(19.8)	[18.3–67.6]	
	Exit	2011	69.6(8.8)	[58.6–80.6]	0.02
	Follow-up	2012	85.8(7.2)	[78.0–93.5]	0.01
	2017 assessment	2017	64.6(17.3)	[43.1–86.0]	0.03
	2019 assessment*	2019	69.2(9.6)	[56.7–92.0]	0.03
**Laboratory group two (*n*=5)**					
	Baseline	2011	29.4(13.0)	[13.1–45.6]	
	Exit	2012	70.6(7.3)	[61.5–79.7]	<0,001
	Follow-up	2013	79.8(4.9)	[73.6–85.9]	0.04
	2014 assessment	2014	82.4(4.7)	[76.5–88.2]	0.01
	2015 assessment	2015	87.2(4.7)	[81.2–93.1]	0,002
	2016 assessment	2016	78.4(6.2)	[70.6–86.1]	0.1
	2017 assessment	2017	71.8(9.7)	[59.7–83.8]	0.8
	2018 assessment	2018	75.8(6.1)	[68.1–83.4]	0.2
	2019 assessment	2019	86.6(5.2)	[74.1–87.0]	0.03
**Laboratory group three(*n*=37)**					
	Baseline	Different years	35.0(7.5)	[32.5–37.5]	
	Exit	Different years	64.7(9.3)	[61.6–67.8]	<0,001
	Follow-up	2019	57.3(9.4)	[53.7–59.5]	<0,001

*The comparison was made for four instead of five laboratories as one laboratory did not undergo 2019 assessment. Mean (SD) for the four laboratories at the follow-up assessment (not mentioned in Table 3) =84.4% (6.3), CI [74.3–94.6] and 69.2% (9.6), CI [56.7–92.0] at the exit assessment.

**Figure 1.**
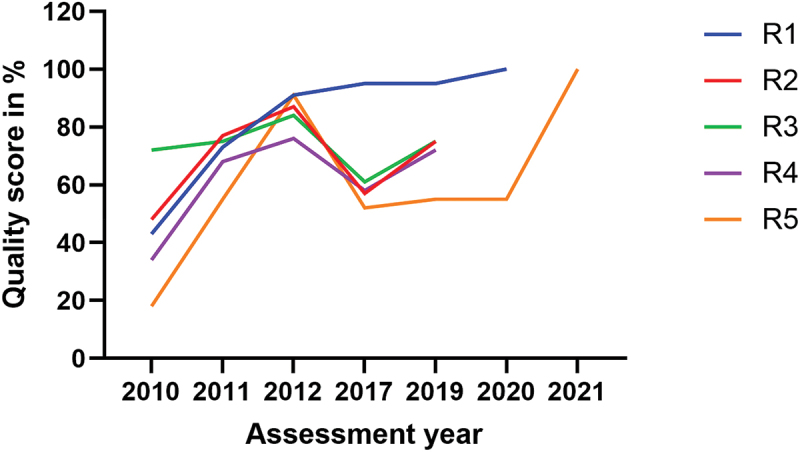
Quality performance in group one laboratories (*n* = 5).

The group one laboratories (R1-R5) enrolled in the SLMTA programme in 2010 and underwent baseline assessments. Initially, these laboratories achieved scores ranging from 18% to 72%, with a mean (SD) score of 43% (19.8). Four out of the five laboratories scored zero stars, with the highest-scoring laboratory, among these four, achieving 48%. Notably, the remaining laboratory in this group achieved a score of 72%, equivalent to a two-star rating. Following these baseline assessments, the laboratories received training in LQMS and benefited from staff mentorship while implementing quality improvement projects. One year later, at the exit assessment, these laboratories demonstrated substantial improvement, with scores ranging from 55% to 77%, with a mean (SD) score of 69.6% (8.8). Four laboratories in group one achieved two to three stars, except one laboratory which secured a one-star rating (55%). In the subsequent follow-up assessment, conducted one year after the exit assessment, these laboratories continued to exhibit higher quality scores, ranging from 76% to 91%, with a mean score of 85.8% (7.2). Ratings ranged between three to four stars (Figure 1).

The difference in quality performance before and after the intervention for group one was significant, with *p* = 0.02 at exit assessment versus baseline, and *p* = 0.01at follow-up versus exit assessment (Table 3). However, it is worth mentioning that four out of the five laboratories in group one experienced a significant decline in performance in 2017, five years later after the follow-up assessment in 2012. In this particular year (2017), one laboratory scored zero stars, three laboratories scored one star, and one laboratory secured five stars. Compared to their follow-up assessments, the decline in quality performance in 2017 and 2019 assessments was significant at *p* = 0.03 in both assessments (Table 3). Interestingly, two laboratories in this group achieved ISO 15,189 standard accreditation, one in 2020 and the other in 2021.

**Figure 2.**
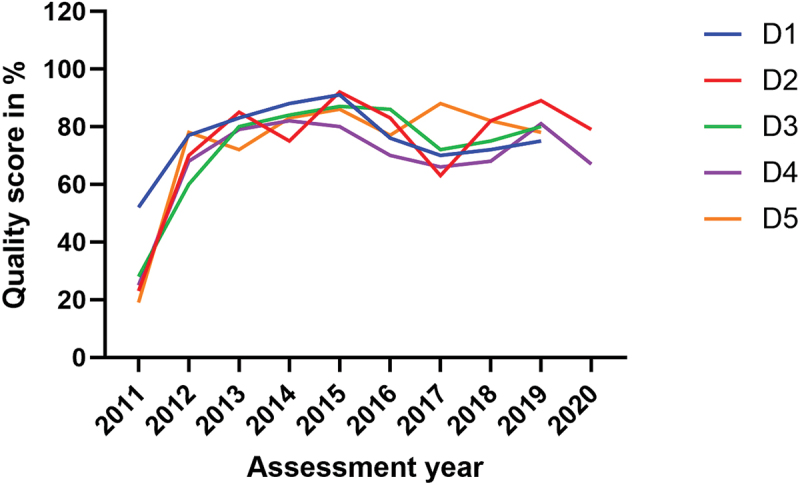
Overtime quality performance in group two laboratories (*n* = 5).

The group two laboratories (D1-D5), all from district hospitals, were enrolled in SLMTA in 2011. These laboratories received continuous mentorship until 2016 and underwent continuous annual assessments with financial incentives based on their performance. Despite their initial baseline scores ranging from 19% to 52%, with a mean (SD) score of 29.4% (13.0) and despite receiving zero stars, these laboratories steadily improved their quality performance, reaching scores between 80% and 92% with a mean (SD) of 87.2% (4.7) in 2015. This high-performance level was maintained until 2019 (Figure 2). Statistically, compared to the baseline, the improvement was significant at exit assessment (*p* < 0.001) like in other two laboratory groups. However, the group two laboratories continued to increase their quality performance in the subsequent assessments, compared to their exit assessments, with significant differences in most of the assessments (Table 3).

**Figure 3.**
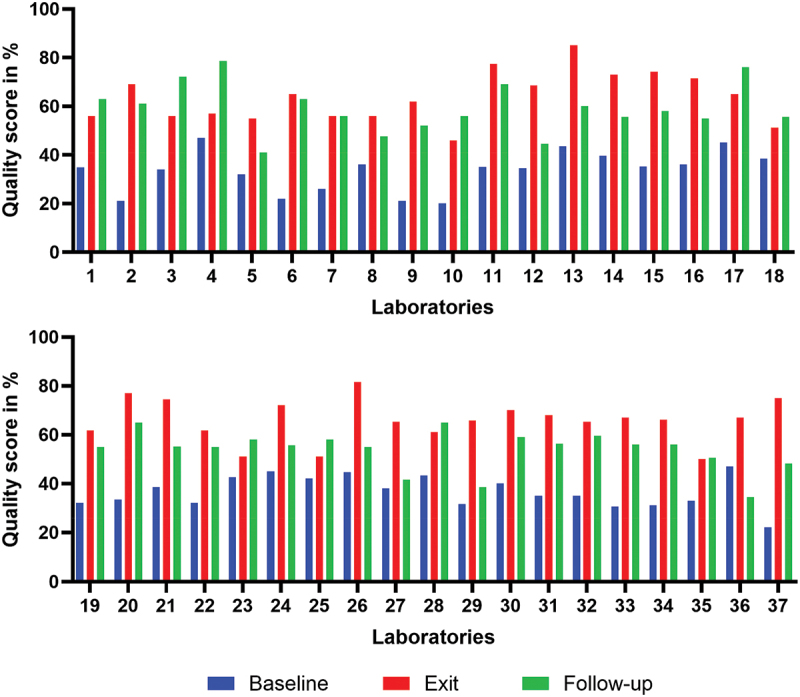
Evolution of quality performance at baseline, exit, and follow-up assessments in group three laboratories (*n*=37).

The group three laboratories (1–37), also based in district hospitals, were enrolled in various cohorts between 2013 and 2017. They achieved baseline scores ranging from 20% to 47%, with a mean (SD) score of 35% (7.5) all of which translated to zero-star ratings. Improvement was evident at the exit assessment, where scores ranged from 46% to 85%, with a mean score of 64.7% (9.3). Statistically, the difference between the two mean scores was highly significant (*p* < 0.001) (Table 3). The star ratings were distributed as follows: zero stars in 13.3% (5 laboratories), one star in 27% (10 laboratories), two stars in 46% (17 laboratories), three stars in 11% (4 laboratories), and four stars in 3% (1 laboratory) (Table 2). Despite this improvement in quality performance at the exit assessment, a significant regression in performance was observed in group three during the follow-up assessment in 2019, following the conclusion of the intervention, with a significant difference between exit and follow-up means (*p* < 0.001). Particularly, as shown in Figure 3, 70% (26 out of 37) of laboratories exhibited a decline in performance. Scores in this assessment ranged from 34% − 79%, with a mean (SD) score of 57.3% (9.4).

In summary, when considering all laboratory groups, the results indicated an improvement in quality performance between baseline and exit assessments following the SLMTA intervention. This improvement increased until the follow-up assessment for group one, as mentorship continued until at this assessment. Most laboratories experienced a decline in performance following the conclusion of the SLMTA intervention in groups one and three. However, the group two laboratories, which continued to receive the intervention for longer period of time, maintained a higher and progressively improving performance.

## Discussion

In this study, we observed the decline in the quality performance of most clinical laboratories in Rwanda after the phase out of the SLMTA programme or similar interventions. This highlighted the issue of sustaining quality laboratory services. This decline had been previously identified in studies conducted in the same settings, which specifically focused only on the five referral laboratories [23,24,28]. The present study extended this concern to the national scale, hence advocating for a solution emphasising a systemic approach. The primary aim of the SLMTA programme is to support national efforts in guiding clinical laboratories in LMICs towards achieving ISO 15,189 standard accreditation and enhancing service quality [21]. Despite the Programme’s demonstrated effectiveness (Figures 1–3; Table 3) [22,29], it is evident that, without thoughtful national strategies to sustain and perpetuate the lessons learned from SLMTA programme for continuous quality improvement, laboratories may regress to their baseline state [23].

The findings of this study, particularly regarding the quality performance observed in group two laboratories (Figure 2 and Table 3), where mentorship and regular annual assessments were maintained, lend support to the hypothesis that these factors can help laboratories sustain the adoption of LQMS and continuous quality improvement. These five laboratories, situated within 4–20 km of national borders, were strengthened in order to contribute to addressing cross-border epidemic infections within the East African community region [26]. Such particular attention, if scaled up to other laboratories (referral and district laboratories), it can enhance quality, as was the case for the group two laboratories.

While the financial incentives provided to the hospitals hosting these specific laboratories within the quality improvement framework deserve mention, it is unlikely that financial incentives alone can explain this superior performance. In comparison to the referral laboratories (Figure 1), which possess greater capacity and resources, including human resources and budget allocations, it is unlikely that the difference in quality performance between the two groups could be attributed solely to financial incentives, but rather to the regular follow-up assessments that identified gaps in LQMS, which are then addressed. Yet, the financial incentive and mentorship programme were phased out in 2015 and 2016, respectively and the group two laboratories continued to exhibit better performance until their final annual assessment in 2019.

Furthermore, the higher quality performance observed in the group two is also evident across all laboratories in this study between the baseline and exit assessments, including those which did not receive additional financial incentives on top of their ordinary budget. The same trend was observed in the group one laboratories, when comparing exit and follow-up assessments, while mentorship was still available. Moreover, we observed an enhanced performance in the 2019 assessment of these referral laboratories, building upon the results of the 2017 assessment and addressing the identified gaps. However, this improvement did not significantly bridge the gap referencing their follow-up assessment performance (Figure 1 and Table 3). The key difference between the group two laboratories and others is that the former had a comprehensive five-year plan for quality improvement and received support to reach their goals [26].

Numerous studies corroborate our findings, particularly regarding demonstrating improvements in laboratory quality, between SLMTA baseline and exit assessments, attributed to personnel training in LQMS, mentorship, and assessments to identify and address gaps [22,29]. However, there are limited publications addressing how laboratories involved in SLMTA sustain their achieved quality performance and progress towards accreditation. A study conducted in Kenya, seven years after the SLMTA phase-out, reported outcomes similar to our study [30]. The interventions of training of personnel in LQMS, mentorship, and assessments, as seen in the group two laboratories’ interventions in our study, also led to positive outcomes in Kenya [30]. A recently published study, collecting data spanning seven years starting in 2013, on medical laboratories implementing quality management systems in Sub-Sahara Africa, indicates that a national framework including laboratory policies and a strategic planning is likely to facilitate ISO 15,189 standard accreditation [31]. These three elements define priorities for laboratories, and similar attention was maintained in the case of the group two laboratories’ mentorship and assessment, yielding improved outcomes.

It is essential to note that this study primarily relied on programme implementation data, resulting in a quasi-experimental design. This design has inherent limitations in establishing a causal association between interventions and outcomes. However, it is important to emphasise that these limitations do not undermine the conclusions drawn from this study within the confines of its design. Notably, data collection was consistently conducted using the same methodology and tool in comparable settings, with the exception of referral laboratories, which are more advanced in terms of operations and capacity.

The SLMTA programme has so far been implemented in 1,643 laboratories across 56 LMICs on various continents. Among these laboratories embarking on the path towards accreditation, 397 have successfully achieved it [32]. Our study shed light on the challenges keeping laboratories in Rwanda from attaining the goal of accreditation and delivering quality services. It also showcased best practices from laboratories that benefited from regular follow-ups resulting in improved performance. Based on these findings, it is essential to underscore that a combination of LQMS knowledge, coupled with effective mentorship, regular assessments, and a national approach, such as a thoughtful strategic plan and policy on laboratory quality improvement, sustains the clinical laboratory quality and propels them toward accreditation. The insights gained from this study can inform the development of strategies to sustain quality and progress toward accreditation, not only in Rwanda but also in other countries of the same context facing similar challenges.

In conclusion, considering these results, the issue of sustaining quality in clinical laboratories in Rwanda, and potentially in other countries with similar settings, remains an issue. Strategies to attain and sustain said quality have been elucidated. The initiation of the SLMTA programme alone is insufficient for the sustained quality of clinical laboratories. Rather, retaining knowledge of LQMS coupled with measures, such as supervision, mentorship, regular assessments to address identified gaps, and the implementation of a coordinated national plan aimed at fostering a culture of quality, are crucial factors in achieving the sustainable quality in clinical laboratories.
